# Precision cardiac targeting: empowering curcumin therapy through smart exosome-mediated drug delivery in myocardial infarction

**DOI:** 10.1093/rb/rbad108

**Published:** 2023-12-13

**Authors:** Ming Chen, Shengnan Wang, Yihuan Chen, Han Shen, Lei Chen, Liang Ding, Qingsong Tang, Ziying Yang, Weiqian Chen, Zhenya Shen

**Affiliations:** Department of Cardiovascular Surgery of the First Affiliated Hospital & Institute for Cardiovascular Science, Suzhou Medical College, Soochow University, Suzhou 215006, China; Department of Cardiovascular Surgery of the First Affiliated Hospital & Institute for Cardiovascular Science, Suzhou Medical College, Soochow University, Suzhou 215006, China; Department of Cardiovascular Surgery of the First Affiliated Hospital & Institute for Cardiovascular Science, Suzhou Medical College, Soochow University, Suzhou 215006, China; Department of Cardiovascular Surgery of the First Affiliated Hospital & Institute for Cardiovascular Science, Suzhou Medical College, Soochow University, Suzhou 215006, China; Department of Cardiovascular Surgery of the First Affiliated Hospital & Institute for Cardiovascular Science, Suzhou Medical College, Soochow University, Suzhou 215006, China; Department of Cardiovascular Surgery of the First Affiliated Hospital & Institute for Cardiovascular Science, Suzhou Medical College, Soochow University, Suzhou 215006, China; Department of Cardiovascular Surgery of the First Affiliated Hospital & Institute for Cardiovascular Science, Suzhou Medical College, Soochow University, Suzhou 215006, China; Department of Cardiovascular Surgery of the First Affiliated Hospital & Institute for Cardiovascular Science, Suzhou Medical College, Soochow University, Suzhou 215006, China; Department of Cardiovascular Surgery of the First Affiliated Hospital & Institute for Cardiovascular Science, Suzhou Medical College, Soochow University, Suzhou 215006, China; Department of Cardiovascular Surgery of the First Affiliated Hospital & Institute for Cardiovascular Science, Suzhou Medical College, Soochow University, Suzhou 215006, China

**Keywords:** myocardial infarction, exosome, targeting, mononuclear phagocyte system, peptide

## Abstract

Nanoparticle-mediated drug delivery has emerged as a highly promising and effective therapeutic approach for addressing myocardial infarction. However, clinical translation tends to be a failure due to low cardiac retention as well as liver and spleen entrapment in previous therapies. Herein, we report a two-step exosome delivery system, which precludes internalization by the mononuclear phagocyte system before the delivery of therapeutic cardiac targeting exosomes (Exo^CTP^). Importantly, curcumin released by Exo^CTP^ diminishes reactive oxygen species over-accumulation in ischemic myocardium, as well as serum levels of lactate dehydrogenase, malonyldialdehyde, superoxide dismutase and glutathione, indicating better antioxidant capacity than free curcumin. Finally, our strategy was proven to greatly potentiate the delivery and therapeutic efficacy of curcumin without systemic toxicity. Taken together, our smart exosome-mediated drug delivery strategy can serve either as therapeutics alone or in combination with other drugs for effective heart targeting and subsequent wound healing.

## Graphical Abstract

**Figure:**
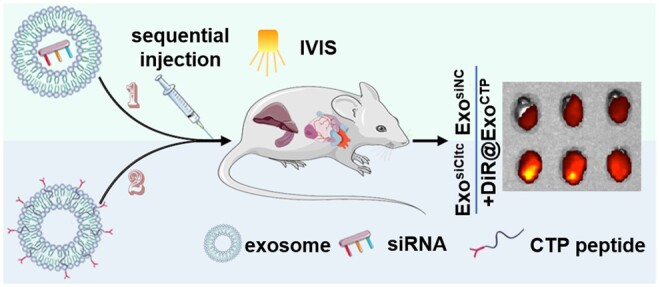

## Introduction

Myocardial infarction (MI) represents a significant global health burden, contributing substantially to illness and death on a global scale [1, 2]. During MI, the sudden and prolonged blockage of the coronary artery hampers an adequate supply of blood and oxygen to the myocardium. This deprivation of essential nutrients leads to over-accumulation of reactive oxygen species (ROS), cellular demise and impaired cardiac function [3–5]. Recently, exosomes have gained significant attention as a promising vehicle for drug delivery in the treatment of cardiac diseases due to their intrinsic biocompatibility and circulation stability [6, 7]. However, recent biodistribution studies of unmodified exosomes validated that very few of them are delivered to the heart via systemic administration [8]. Therefore, an efficient and safe delivery system is critically needed for therapies against MI.

Past studies have focused on enhancing delivery efficiency to cardiomyocytes using targeted peptides [9]. To offset off-target delivery, we previously developed ischemic heart-targeting exosomes with lentivirus over-expressing cardiac targeting peptide (CSTSMLKAC, CTP). These CTP exosomes are localized more specifically to the ischemic myocardium and offer cardio-protection during acute MI [10]. However, due to biosafety concerns, these aforementioned virus-based interventions are still unmet for translational medicine.

Besides limited organ targeting capability, rapid internalization of exosomes by the mononuclear phagocyte system (MPS) presents a hurdle for effective drug delivery to the heart. The effectiveness of therapeutic exosomes is hampered by entrapment primarily in the liver and spleen, both rich in MPS activity [11]. Hence, it is crucial to address this issue of non-specific uptake by MPS-dominant organs to improve exosome delivery efficiency to the ischemic heart.

Macrophages can internalize exosomes using multiple mechanisms, including Clathrin heavy chain 1 (Cltc)-dependent endocytosis, micropinocytosis, phagocytosis and fusion with the plasma membrane [12]. Clathrin is a crucial protein involved in the process of generating vesicles with a coated structure. It forms a triskelion architecture comprising three light chains and three heavy chains, which self-assembles to facilitate the budding process of endocytosis [13]. Previous studies have documented that inhibitors like dynasore and chlorpromazine, which target Cltc-dependent endocytosis, effectively hinder exosome internalization by macrophages [14].

Curcumin, the active compound found in turmeric, is thought to possess beneficial properties that can safeguard the cardiovascular system [15]. Despite its therapeutic potential for MI treatment, its effectiveness is hindered by poor tissue bioavailability caused by its low stability, solubility and rapid systemic elimination [16]. Hence, encapsulating curcumin in exosomes may be an effective drug delivery strategy to overcome these issues.

In our present study, we first decorated mesenchymal stem cell (MSC)-derived exosomes with DOPE-PEG-CTP to enable their selective uptake by cardiomyocytes. To alleviate entrapment by the liver and spleen, we established a two-step drug delivery system, which pre-blocks their uptake before delivery of therapeutic exosomes. Finally, our strategy was proven to greatly potentiate the therapeutic efficacy of curcumin without systemic toxicity. In summary, we have designed a smart drug delivery system aimed at encouraging direct heart targeting while blocking passive MPS endocytosis. This approach holds promise for advancing cardiac healing in the future.

## Materials and methods

### Cell culture and hypoxic treatment

MSC cultures derived from C57BL/6 bone marrow (Cyagen Biosciences) were maintained using mesenchymal stem cell growth medium (MUXMX-80011, Cyagen Biosciences), following previously established protocols [17]. Differentiation of MSCs into adipocytes, osteocytes and chondrocytes was confirmed with respective kits (Cyagen Biosciences). HL-1 (Otwo Biotech) and MCEC cells were cultured in DMEM/HIGH Glucose medium (Procell) supplemented with 10% fetal bovine serum (FBS, Excell Bio) and 1% penicillin–streptomycin (60162ES76, Yeasen). To mimic MI injury, HL-1 cells were hypoxic preconditioned (1% O_2_) for 12 h.

### Exosome isolation, qualification and characterization

FBS was subjected to centrifugation at 110 000 *g* for 8 h to remove exosomes. MSCs were then cultivated using exosome-depleted FBS. MSC-derived exosomes were isolated from the collected supernatant following established methodologies [18–20]. Briefly, the culture supernatant underwent a series of centrifugation steps to eliminate cellular debris, that was 300 *g* for 10 min, 3000 *g* for 20 min, and finally 10 000 *g* for 30 min. To further refine the sample, it was subsequently filtered using a 0.22 µm filter. The filtrate was finally centrifuged twice at 110 000 *g* for 90 min. Pelleted exosomes were resuspended in PBS and analyzed using a BCA Protein Assay Kit (WB6501, New Cell & Molecular Biotech). Morphology of ultracentrifugated exosomes (by Wuhan MISP Bio-technology) was identified using transmission electron microscopy (TEM) (JEOLJEM-2100) [21, 22] and atomic force microscopy (AFM) (Dimension Icon). Nanoparticle tracking analysis (NTA) was assessed with the ZetaView instrument (Particle Metrix) by VivaCell Shanghai Biosciences Co., Ltd. The zeta potential and polydispersity index (PDI) were assessed using dynamic light scattering (DLS) (Brookhaven).

### Ligand conjugation to exosomes

Exosomes were labeled with either a Cardiac Homing Peptide (CTP, CSTSMLKAC), which is known to specifically target the infarct heart, or a Scramble peptide (Scr, CSKTALSMC), which has the same chemical composition but contains a randomized sequence [23]. Conjugation of peptides to exosomes was achieved by employing established methods [24]. Briefly, DOPE-NHS (Ruixibio) and peptides were mixed with a 100-fold molar excess of the peptide and allowed to undergo a 1 h reaction, resulting in the formation of DOPE-peptide complexes. Subsequently, the DOPE-peptide complexes were incubated with exosomes at a lipid-to-exosome ratio of 6000. Modified exosomes were analyzed using a BCA Protein Assay Kit.

### siRNA loading into exosomes

To load exosomes with siCltc (GenePharma), electroporation was performed using a 4D-Nucleofector System (Lonza), following the methodology outlined in our previous study [25]. After removing the free-floating siRNAs with RNase A (CoWin Biosciences) and RNase inhibitor (E125, Novoprotein), exosomes were purified. The sequence for siCltc (mus) oligonucleotides is provided below: 5′-GCUCAUCAAUGUUUGCAAUTT-3′.

### Curcumin incorporation into exosomes

Curcumin dissolved in DMSO (M3850, AbMole, USA) was mixed with exosomes at room temperature. To remove any unbound curcumin, the mixture underwent two rounds of centrifugation at 5000 *g*, followed by ultracentrifugation at 110 000 *g* for 3 h, following the established protocol [26].

### NanoView microarray for surface marker characterization

Debris-depleted culture medium was collected and dropped on microarray chips as described in Wang *et al*. [27]. Three chip spots were each precoated with capture antibodies against CD81 and CD9, and negative controls. The chips were then incubated with detection antibodies including anti-CD9-AF488, anti-CD81-AF555 and anti-CD63-AF647. Chips were finally imaged by ExoView R100 (NanoView Biosciences) and data were analyzed using off-line ExoViewer3 EAP software.

### In vivo biodistribution of exosomes

MI injury was induced by ligating the left anterior descending coronary artery, which was verified by color change in the left ventricle. On the 2nd day, DiR-labeled Exo^Scr^ or Exo^CTP^ was intravenously injected through the tail vein [28]. DiR fluorescence distribution in the heart, liver, spleen, lung and kidney was further imaged by *in vivo* spectrum imaging system (PerkinElmer) 6 h post-injection [29–31].

### Internalization of exosomes *in vivo* and *in vitro*

To determine *in vivo* uptake, DiI-labeled exosomes were intravenously injected and hearts were sliced up 24 h post-injection. The sections were immunofluorescent-stained with antibodies against Cardiac Troponin T (cTnT), CD31 or vimentin (Abcam), and then counter-stained with DAPI-containing anti-fading solution (S2110, Solarbio) as described previously [32]. To evaluate *in vitro* uptake, DiI-labeled exosomes were incubated with HL-1 cells for 24 h, and internalization was visualized by confocal microscopy (Zeiss, LSM880).

### Exosome dynamic uptake imaging

Dil-labeled Exo^Scr^ or Exo^CTP^ was added to HL-1 cells, and the culture dish was placed in a living cell workstation. The captured cells were observed and visualized at 5-min intervals over a period of 6 h using a confocal microscope (Zeiss, LSM880).

### Transwell co-culture system

To investigate the transmigration ability of Exo^CTP^, a transwell co-culture assay was conducted [18]. Briefly, 1 × 10^5^ MCEC and HL-1 cells were seeded in membrane insert (0.4 μm) and basolateral chamber, respectively. After 12 h of hypoxic treatment, 10 μg/ml DiI-labeled Exo^CTP^ or Exo^Scr^ was added into the upper chamber. After 12 h of incubation, HL-1 cells with DiI signal were observed under a fluorescence microscope.

### In vivo safety evaluation

To calculate short-term *in vivo* biological safety, mice were administered with free curcumin, Exo^CTP^ and Cur@Exo^CTP^ on Days 0, 1, 2 and 7 via tail vein injection, and serum was collected on Days 3 and 8. Alanine aminotransferase (ALT), aspartate aminotransferase (AST), blood urea nitrogen (BUN) and creatinine (CRE) were detected with respective detection kits (BC1555, BC1565, BC1535 for Solarbio or F10315 for Westang Biotech) as described previously [28, 33]. To investigate long-term *in vivo* biosafety, major organs, including the liver, kidney, lung, spleen and brain were H&E-stained for morphology assay on Day 28 post-injection [34, 35].

### Cardiac protection efficiency evaluation

Animal welfare and experimental procedures were approved by the Ethics Committee of Soochow University (SUDA20230402A01). Each mouse underwent transthoracic echocardiography on Days 0, 3, 7, 14 and 28 under anesthesia. Cardiac function was evaluated via M-mode imaging and the analysis of left ventricular ejection fraction (LVEF) and fractional shortening (FS) was conducted following uniform standards [36]. Subsequently, mouse hearts were sliced into 5-μm sections and the infarct area was visualized and quantified using Masson's staining (KeyGEN BioTECH).

### Measurement of ROS in tissue sections

To assess ROS accumulation in the myocardium, frozen tissue sections were treated with 5 μmol/L dihydroethidium (DHE, ID3560, Solarbio). The resulting images were captured using an inverted fluorescence microscope (Olympus), and fluorescence signals were quantified using Image J software. Fluorescence intensity in the experimental group was finally normalized to that of the PBS group.

### Detection of oxidative parameters

Serum SOD, MDA and LDH levels were assayed using commercial kits (Jiancheng). The optical densities were measured using a multifunctional microplate reader (BIO-TEK).

### Flow cytometry

For flow cytometry, PBS-containing 2% BSA (Yeasen) was used for antibody staining. Antibodies including anti-Sca-1-APC, anti-CD29-AF488, anti-CD44-APC, anti-CD11b-PE, anti-CD45-PE, anti-CD117-APC (BioLegend or eBioscience) and corresponding fluorescent conjugated isotype controls (MultiSciences) were used. Flow cytometry was performed on Millipore Guava easyCyte as described previously [36].

### Statistical analysis

The data were expressed as mean ± SD. Statistical analyses were conducted using GraphPad Prism. Two-tailed unpaired Student's *t*-test was employed to compare any two groups. For comparisons involving more than two groups, one-way ANOVA with Tukey’s correction was applied. Multiple comparisons with two independent variables were assessed by two-way ANOVA followed by Tukey’s correction. Statistical significance was defined as a *P* value less than 0.05.

## Results and discussion

### Preparation and characterization of Exo^CTP^

**Figure 1.**
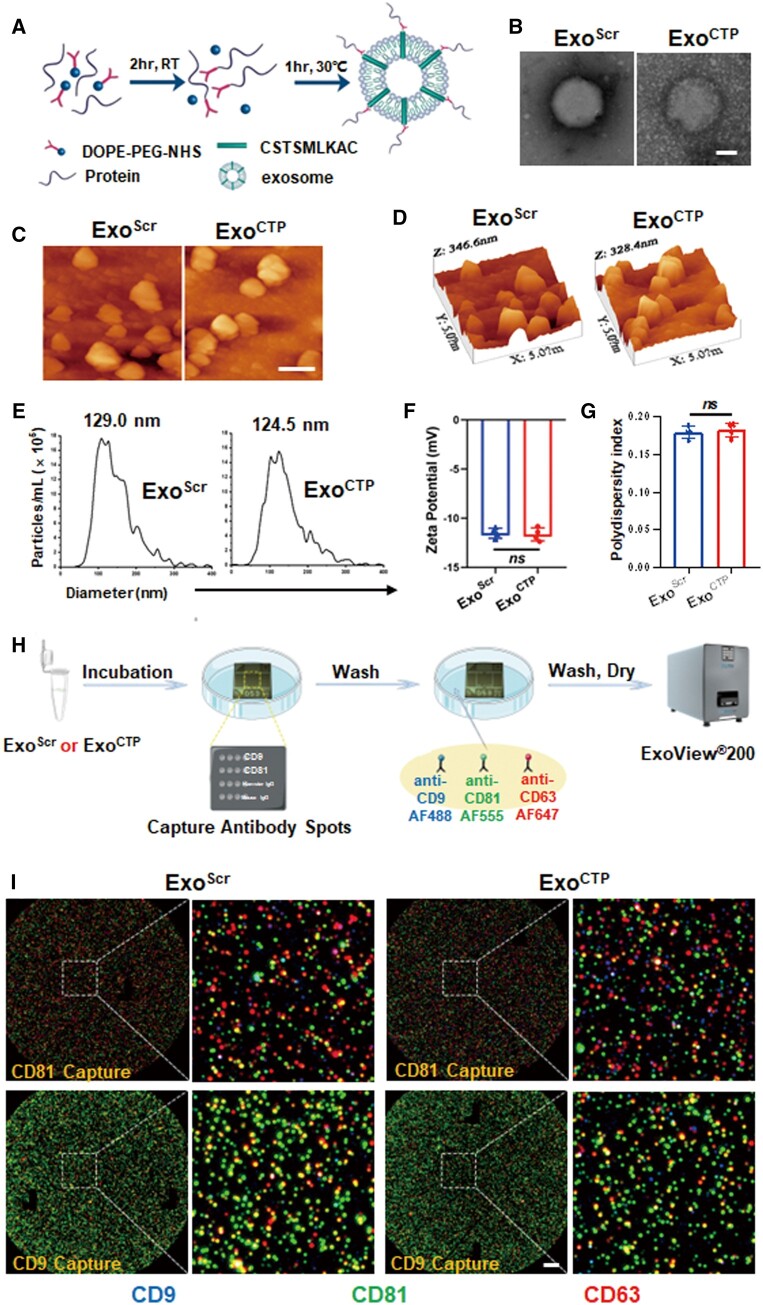
Modification and characterization of Exo^CTP^. (**A**) Schematic diagram of conjugating CSTSMLKAC to exosomes by a two-step reaction. (**B**) Transmission electron micrograph of Exo^Scr^ and Exo^CTP^. Scale bar, 50 nm. (**C**, **D**) Atomic force microscopy images of Exo^Scr^ and Exo^CTP^. Scale bar, 200 nm. (**E**) Particle size distribution determined by nanoparticle tracking analysis (NTA). (**F**, **G**) Zeta potential and polydispersity index (PDI) of Exo^Scr^ and Exo^CTP^ were determined using dynamic light scattering (*n *=* *4). (**H**, **I**) Immunocolocation of exosomes was performed by NanoView microarray. The chips were coated with antibodies against anti-mouse CD81 and CD9 individually. To determine the co-localization, fluorescently labeled antibodies against CD9, CD81 and CD63 were employed. Scale bar, 10 μm. All data are represented as mean ± SD. Two-tailed unpaired Student’s *t* test was utilized. *ns*: not significant.

Mouse MSCs derived from bone marrow were cultured, and exosomes were obtained from the conditioned medium through ultracentrifugation. MSCs homogeneously expressed Sca-1, CD29 and CD44, while lacking CD11b, CD45 and CD117 (Supplementary Figure S1). They also exhibited excellent adipogenic, osteogenic and chondrogenic differentiation potential (Supplementary Figure S2). To conjugate exosomes with protein peptides, a two-step reaction was proposed, generating Exo^Scr^ and Exo^CTP^ (Figure 1A). First, the morphology of exosomes was evaluated using techniques such as TEM and AFM. Not surprisingly, Exo^CTP^ had a typical round cup-shaped structure and was well dispersed (Figure 1B–D), with an average height of 153.4 nm by AFM (Supplementary Figure S3) and a size peaking at 124.5 nm by NTA (Figure 1E). Second, DLS analysis revealed Exo^CTP^ with an average zeta potential distributed −11.64 ± 0.66 mV (Figure 1F) and PDI ranging 0.1825 ± 0.0096 (Figure 1G). Finally, immuno-colocalization chip indicated that both exosomes were positive for common biomarkers; nevertheless, CD63/CD81/CD9 tri-colocalization was not frequently observed (Figure 1H and I), whose detailed mechanisms need to be further investigated.

### Exo^CTP^ initiates active targeting to infarcted hearts

**Figure 2.**
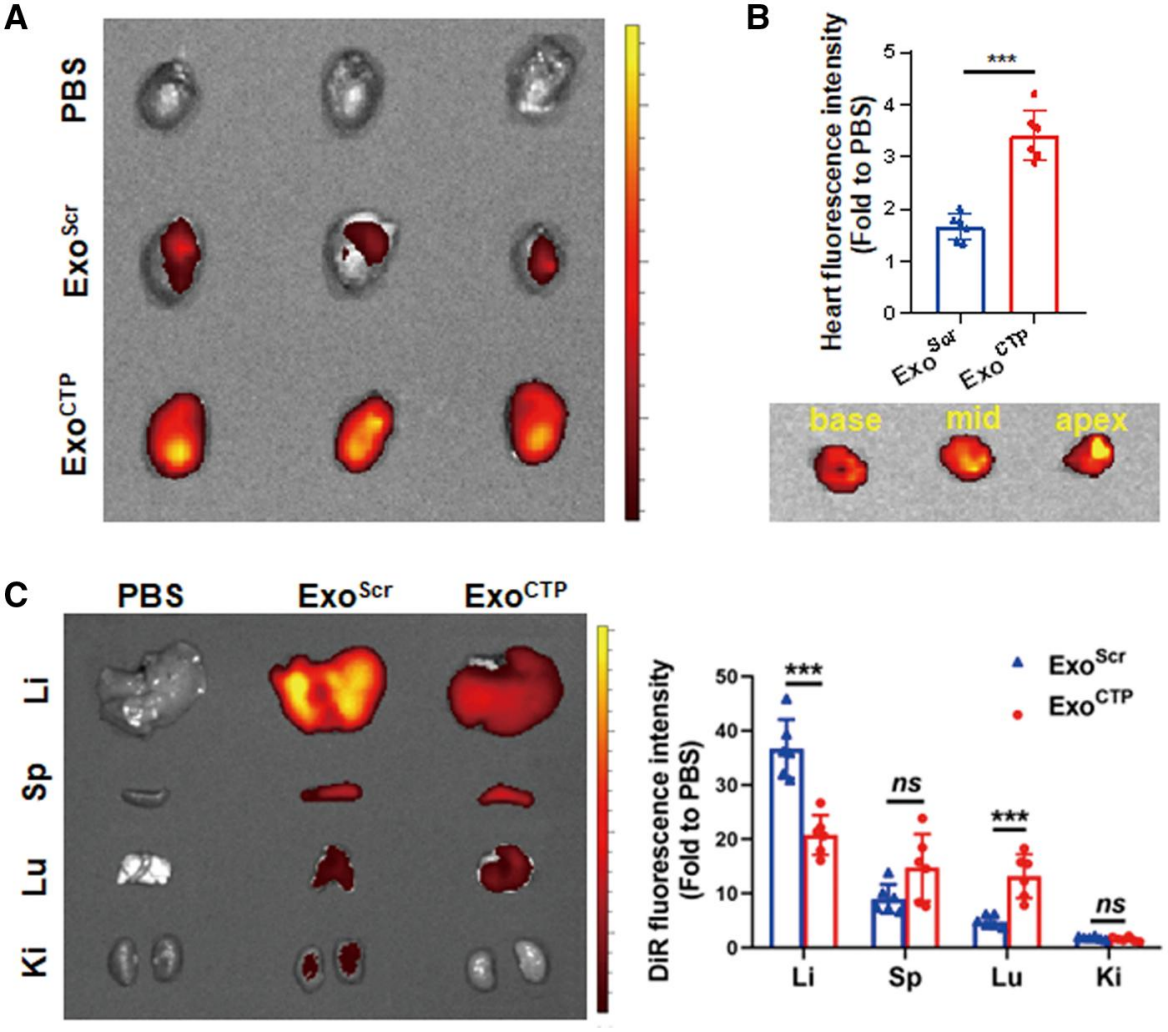
*In vivo* biodistribution of Exo^CTP^. MI mice were intravenously injected with DiR-labeled Exo^CTP^ for *in vivo* tracking. Different organs were dissected 6 h post-injection (**A**) *ex vivo* optical imaging and semi-quantification for infarcted hearts (*n *=* *6). (**B**) *Ex vivo* imaging of heart sections from base to apex in Exo^CTP^ group. (**C**) *Ex vivo* optical imaging and semi-quantification of other major organs (*n *=* *6). All data are represented as mean ± SD. Two-tailed unpaired Student’s *t* test was utilized. *ns*: not significant, ***P < 0.001.

To assess *in vivo* biodistribution of Exo^CTP^, MI mice were injected with DiR-labeled Exo^Scr^ or Exo^CTP^ via the tail vein. Multiple organs, including the heart, liver (Li), spleen (Sp), lung (Lu) and kidney (Ki), were extracted for quantitative DiR fluorescence analysis after 6 h. As expected, DiR signal in Exo^CTP^-injected hearts was 2.059-fold as high as that of Exo^Scr^-injected hearts (*P* < 0.001, Figure 2A). Of note, Exo^CTP^ primarily accumulated in the anterior region of the left ventricle, particularly in areas characterized by ischemia and necrosis (Figure 2B). Additionally, liver, spleen and lung remained the major organs for exosome entrapment (Figure 2C). In sum, these results indicated that compared to Exo^Scr^, Exo^CTP^ accumulated mainly in ischemic hearts.

### Exo^CTP^ directly targets cardiomyocytes in injured hearts

**Figure 3.**
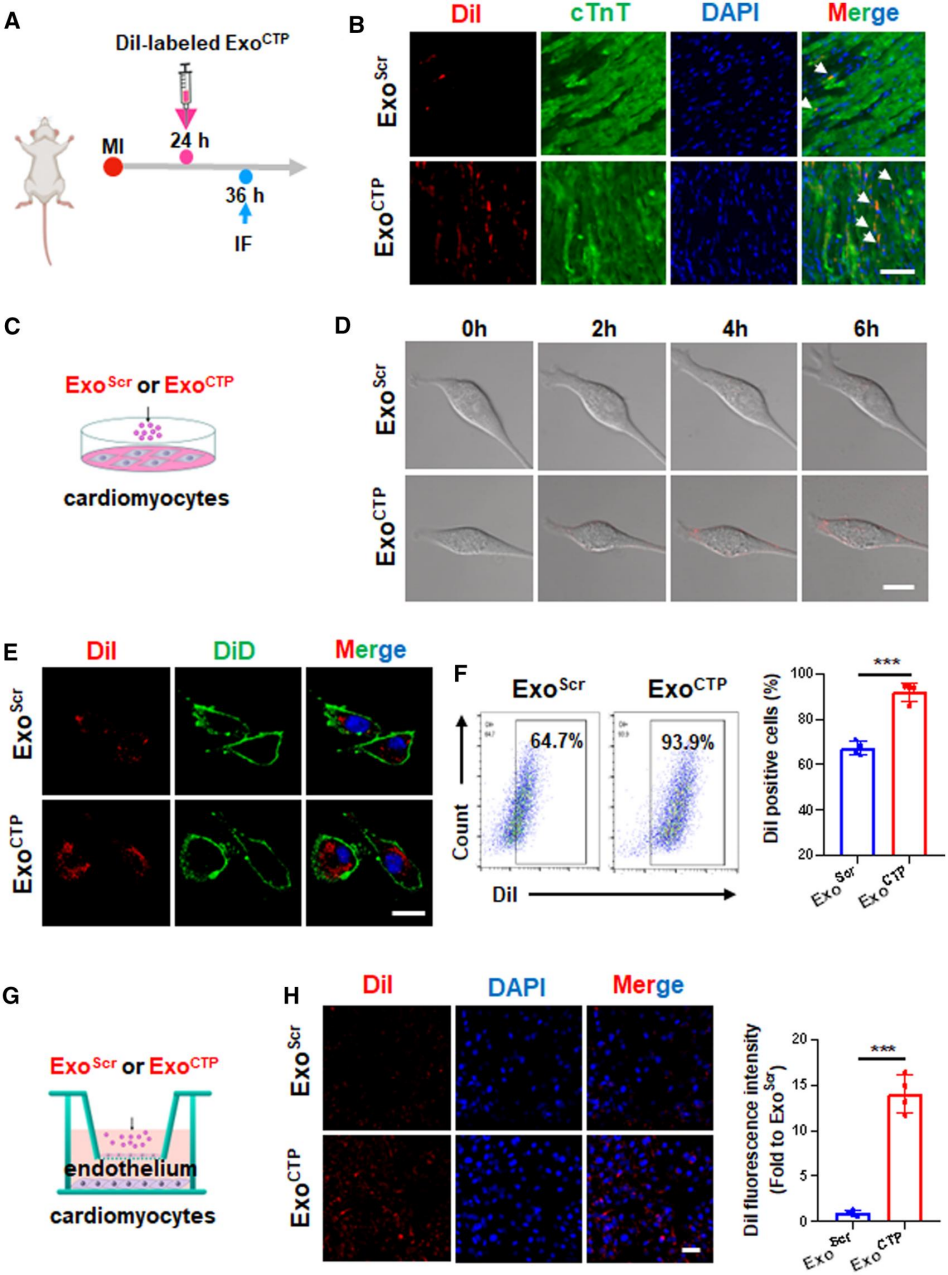
Exo^CTP^ directly targets cardiomyocytes. (**A**) Schematic diagram of *in vivo* Exo^CTP^ tracing. (**B**) Representative section images of DiI-labeled Exo^Scr^ or Exo^CTP^ taken up by cTnT^+^ cardiomyocytes. Scale bar, 50 μm. (**C**) Schematic diagram of *in vitro* Exo^CTP^ tracing. Hypoxic HL-1 cells were incubated with DiI-labeled Exo^Scr^ or Exo^CTP^. (**D**) Bright-field images at 0, 2, 4 and 6 h. Scale bar, 20 μm. (**E**) Immunofluorescent images with DiD-labeled cell membrane illustrating exosome absorption. Scale bar, 20 μm. (**F**) FACS dot plots for DiI signal in HL-1 cells (*n *=* *4). (**G**, **H**) MCECs were plated on membrane inserts, while HL-1 cells were seeded at the bottom of the culture wells before hypoxic challenge. Representative microscopy images and quantification for DiI-labeled Exo^CTP^ uptake (*n *=* *4). Scale bar, 50 μm. All data are represented as mean ± SD. Two-tailed unpaired Student’s *t* test was utilized. ***P < 0.001.

Next, the cellular distribution of Exo^CTP^ was further evaluated both *in vivo* and *in vitro*. For *in vivo* tracing, immunofluorescence staining revealed a higher accumulation of DiI-labeled Exo^CTP^ in cardiomyocytes compared to endothelial cells or cardiac fibroblasts (Figure 3A and B and Supplementary Figure S4). For *in vitro* tracing, DiI-labeled Exo^CTP^ was incubated with HL-1 murine cardiomyocytes. Both dynamic live cell imaging and immunofluorescence analysis indicated that Exo^CTP^ was more efficiently uptaken by target cells (Figure 3C–E). Likewise, flow cytometry analysis further supported enhanced internalization of Exo^CTP^ by HL-1 cells (92.13 ± 4.10% vs. 67.63 ± 3.06%, *P* < 0.001, Figure 3F). To investigate whether Exo^CTP^ can cross blood vessels while targeting cardiomyocytes, an *in vitro* transwell co-culture system was established. As illustrated in Figure 3G, murine cardiac endothelial MCEC cells were seeded on membrane insert, whereas HL-1 cells and DiI-labeled Exo^CTP^ in lower and apical chambers of the transwell, respectively. The exosome transmigrating across the endothelial layer was determined by DiI signal in HL-1 cells. Compared to Exo^Scr^, a 13.03-fold higher DiI intensity was observed in HL-1 cells cocultured with Exo^CTP^ (*P* < 0.001, Figure 3H). Taken together, our results indicated that Exo^CTP^ can efficiently penetrate blood vessels and essentially target and be internalized into cardiomyocytes.

### *In vivo* safety and immunogenicity of Cur@Exo^CTP^

**Figure 4.**
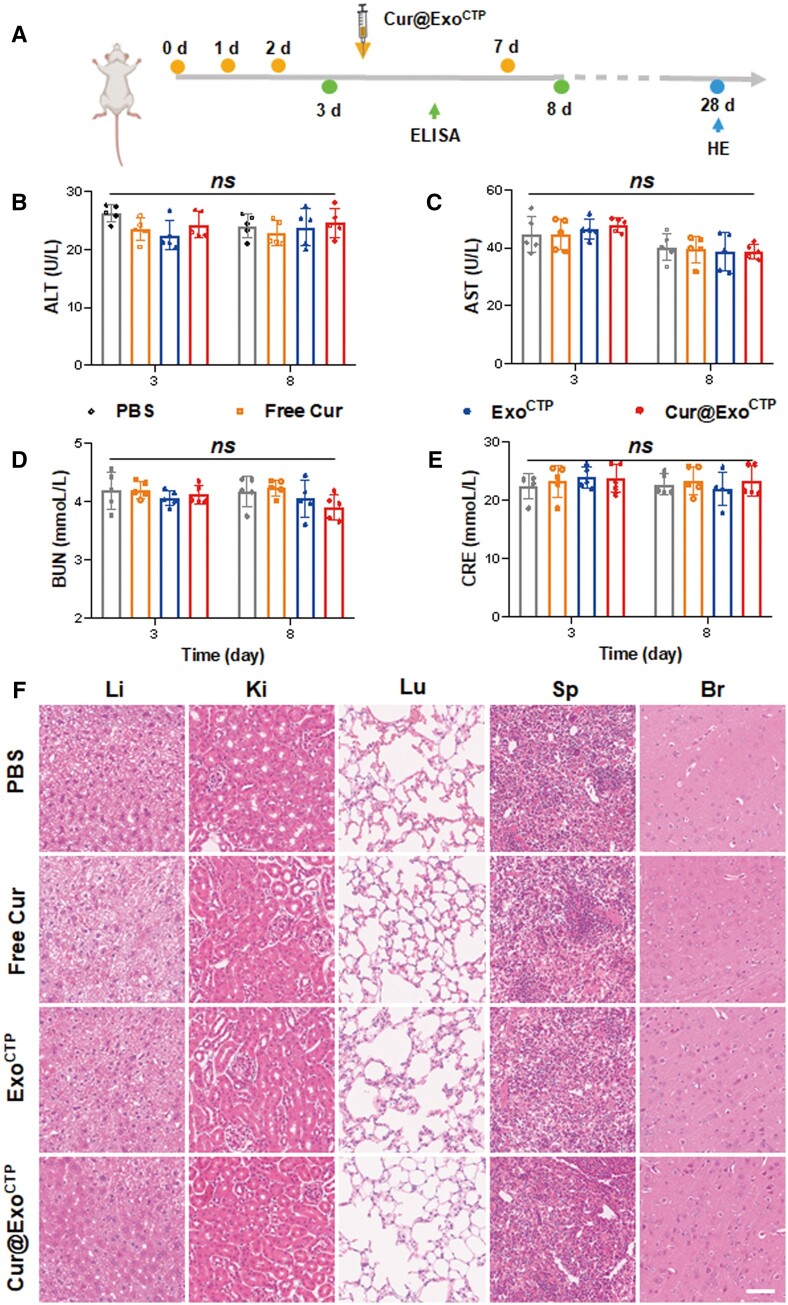
Safety verification of cur@Exo^CTP^. (**A**) Schematic diagram of *in vivo* safety assay. (**B–E**) Serum levels of ALT, AST, BUN and CRE after administration with PBS, free Cur, Exo^CTP^ and Cur@ExoCTP (*n *=* *5). (**F**) H&E staining of major organs showing no obvious histological changes after indicated treatments. Scale bar, 100 μm. ALT, alanine aminotransferase; AST, aspartate aminotransferase; BUN, blood urea nitrogen; CRE, creatinine. All data are represented as mean ± SD. Two-way ANOVA followed with Tukey’s post hoc test was utilized. *ns*: not significant.

Curcumin, being hydrophobic, exhibits limited solubility in water and is highly prone to hydrolysis when exposed to aqueous solutions [13, 37]. We therefore encapsulated curcumin into Exo^CTP^, generating Cur@Exo^CTP^. First, biosafety and immunogenicity of Cur@Exo^CTP^ were evaluated after systemic injection to assess their clinical translational potential (Figure 4A). In terms of short-term biocompatibility, hematological parameter analysis illustrated normal levels of ALT [38], AST, BUN and CRE, indicating unaffected liver and kidney functions by Cur@Exo^CTP^ treatment (Figure 4B–E). For long-term biocompatibility, histopathology staining indicated unaltered tissue structure, cellular morphology, and rare immune cell infiltration in vital organs (Figure 4F). Overall, Cur@Exo^CTP^ exhibited great *in vivo* biosafety in mice.

### Cur@Exo^CTP^ accelerates post-MI cardiac recovery

**Figure 5.**
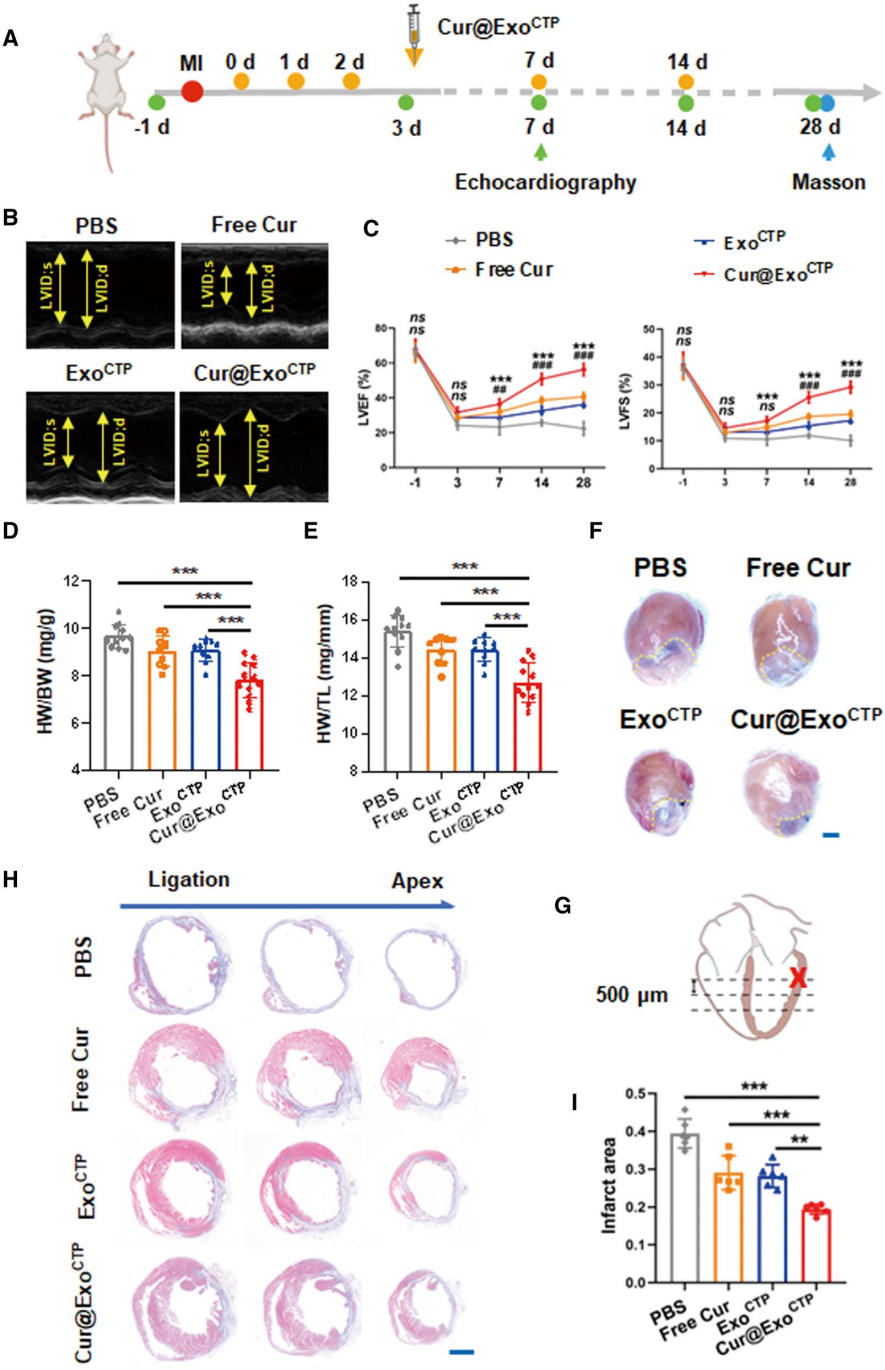
Cur@Exo^CTP^ diminishes post-MI heart dysfunction and infarct size. (**A**) Schematic diagram of experimental time points. (**B**) Representative M-mode echocardiograms on Day 28. (**C**) Left ventricular (LV) EF and FS measured on Days −1, 3, 7, 14 and 28 post-treatment (*n *=* *9–12, * for comparison between Cur@Exo^CTP^ and Exo^CTP^ group, # for comparison between Cur@Exo^CTP^ and free Cur group). (**D–F**) Heart weight normalized to body weight or tibia length and the overall appearance of the heart on Day 28 after MI (*n *=* *10–12). Scale bar, 2 mm. (**G**) Schematic representation indicating ligation (cross) and sampling (dashed line) site. Serial sectioning was conducted at intervals of 500 μm. (**H**, **I**) Masson’s staining images and quantification of infarct area on Day 28 (*n *=* *6). Scale bar, 2 mm. All data are represented as mean ± SD. Either two-way ANOVA with Tukey's post hoc test or one-way ANOVA with Tukey's post hoc test was employed for the statistical analysis. *ns*: not significant, **/^##^*P* < 0.01, ***/^###^*P* < 0.001.

To expand upon our *in vitro* discoveries, we subsequently investigated the potential of Cur@Exo^CTP^ infusion to mitigate the deterioration of MI-induced cardiac function in mice. Left anterior descending artery (LAD)-ligated mice were infused with indicated exosomes on Days 0, 1, 2, 7 and 14 post-surgery (Figure 5A). While Exo^CTP^ alone demonstrated post-MI cardio-protective properties, probably attributed to its intrinsic components, Cur@Exo^CTP^-treated mice did exhibit greatest heart function (Figure 5B and C), as well as least pathologic cardiac dilation (Figure 5D–F). Furthermore, Masson’s Trichrome staining revealed a reduction in fibrotic tissue, indicating myocardial restoration and reduced ischemic area in Cur@Exo^CTP^-treated hearts (Figure 5G–I). Collectively, our results suggested that Cur@Exo^CTP^ therapy effectively ameliorates post-MI heart dysfunction and scar formation.

### Cur@Exo^CTP^ alleviates excessive myocardial oxidative stress

**Figure 6.**
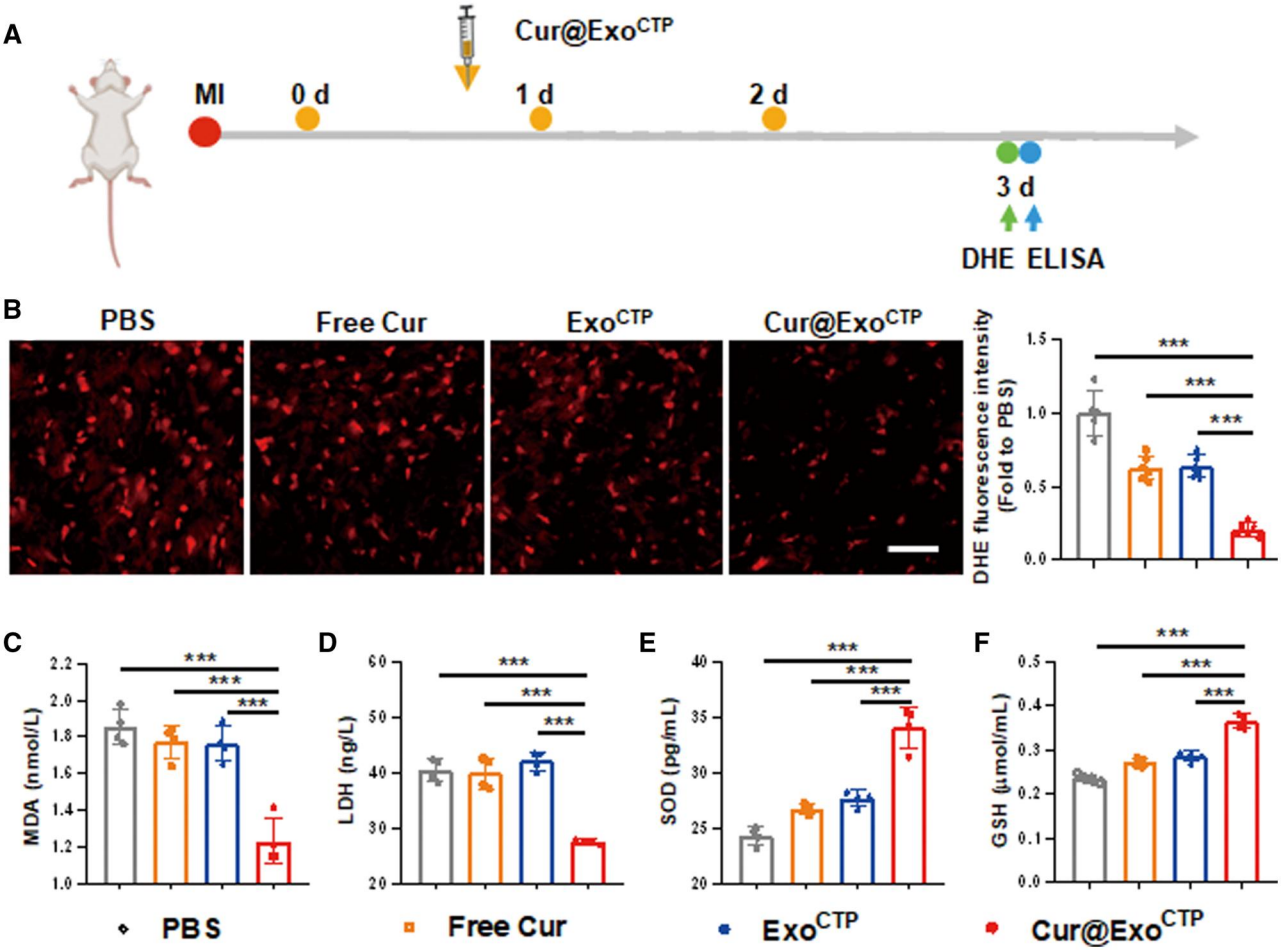
Cur@Exo^CTP^ administration attenuates myocardial oxidative stress. (**A**) Study design for *in vivo* oxidative stress evaluation. (**B**) Representative DHE staining images and quantification (*n *=* *5–6). Scale bar, 50 μm. (**C–F**) Serum level of MDA, LDH, SOD and GSH 12 h after treatment with PBS, free Cur, Exo^CTP^, and Cur@Exo^CTP^ (*n *=* *4). MDA, malonyldialdehyde; LDH, lactate dehydrogenase; SOD, superoxide dismutase; GSH, glutathione. All data are represented as mean ± SD. One-way ANOVA followed with Tukey’s post hoc test was utilized. ***P < 0.001.

Excessive ROS generation is believed to be the dominant source of ischemic injury and may contribute to complex pathophysiological events [39]. In light of this, we quantified myocardial $O_{2}^{-}$ content using DHE staining in ischemia-challenged hearts. MI mice were administered with Cur@Exo^CTP^ through tail vein injection, and heart and serum were harvested for histological and immunochemical analysis 3 days post-therapy (Figure 6A). Importantly, Exo^CTP^ markedly abrogated MI-induced ROS over-accumulation in peri-infarct zones, and this anti-oxidative effect was more pronounced in Cur@Exo^CTP^ group (Figure 6B), indicating a favorable environment for cardiac recovery. Besides, indicators of oxidative stress, including malonyldialdehyde (MDA) production, lactate dehydrogenase (LDH) release, superoxide dismutase (SOD) activity and glutathione (GSH) level were also analyzed. Consistently, Cur@Exo^CTP^ therapy effectively impaired serum levels of LDH and MDA (Figure 6C and D), whereas restored SOD and GSH levels (Figure 6E and F). Taken together, these data provide evidence of diminished ROS generation in ischemic hearts following Cur@Exo^CTP^ therapy.

### Prior injection of Exo^siCltc^ blocks passive entrapment of Exo^CTP^ by liver and spleen

**Figure 7.**
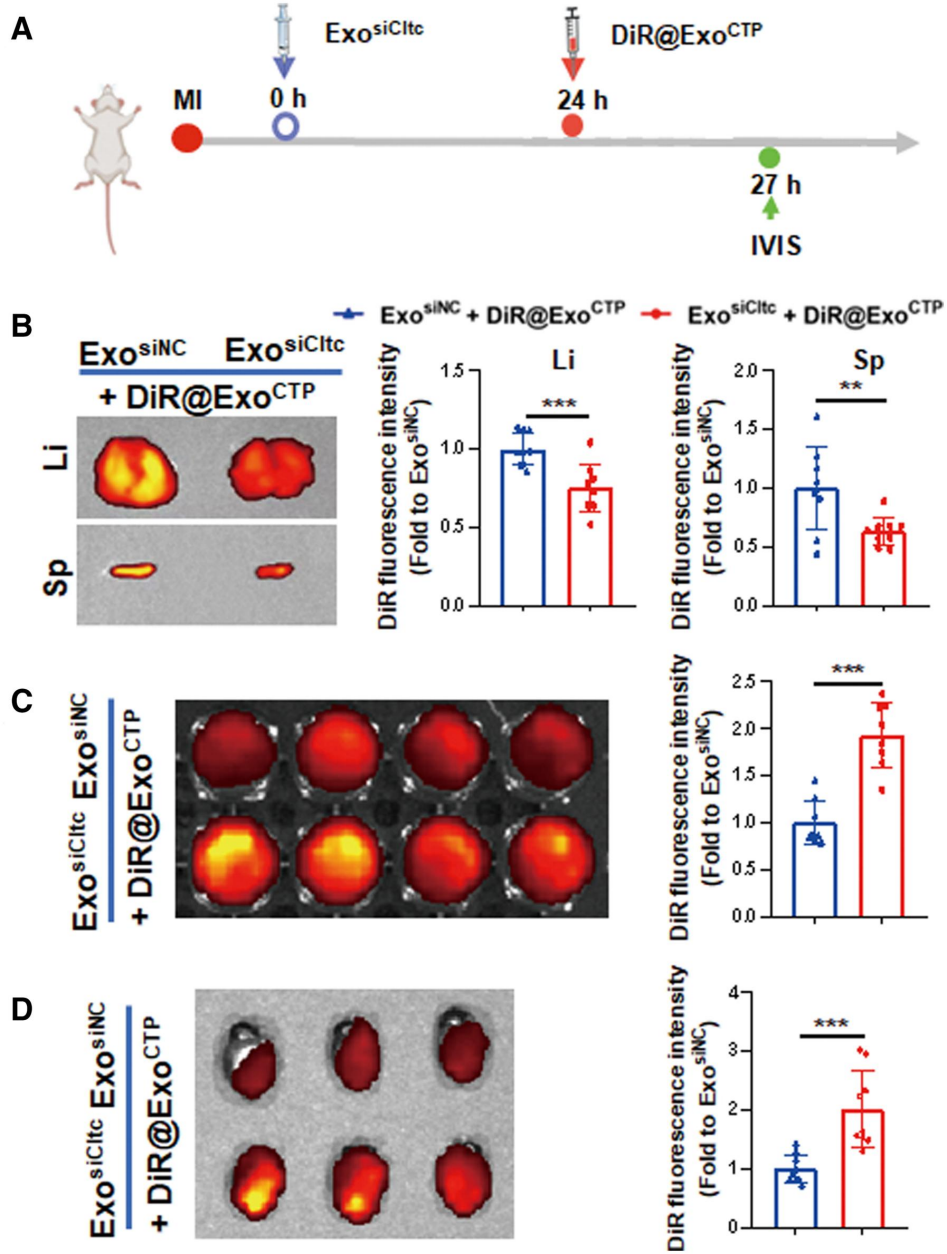
Exo^siCltc^ pre-treatment retards entrapment of Exo^CTP^ by the liver and spleen. (**A**) Schematic diagram for calculating *in vivo* biodistribution of therapeutic Exo^CTP^. Mice were sequentially treated with Exo^siNC^ or Exo^siCltc^ and DiR-labeled Exo^CTP^ for tracking. (**B**) *Ex vivo* fluorescence imaging and quantification of DiR signal in liver and spleen (*n *=* *9). (**C**) *Ex vivo* imaging and quantification of DiR signal in peripheral blood (*n *=* *8). (**D**) *Ex vivo* fluorescence imaging and quantification of DiR signal in hearts (*n *=* *9). All data are represented as mean ± SD. Two-tailed unpaired Student’s *t* test was utilized. **P < 0.01, ***P < 0.001.

In agreement with a previous study [40], Exo^CTP^ was largely entrapped by the liver and spleen (Figure 2C). To alleviate endocytosis by these MPS-heavy organs, we established a two-step drug delivery strategy, which blocked therapeutic exosome uptake by prior knockdown of Cltc *in vivo*. We initially confirmed that Cltc silencing did not alter the tissue architecture, cellular arrangement or immune cell penetration in the liver, kidney, lung, spleen and brain (Supplementary Figure S5). Subsequently, as depicted in Figure 7A, Exo^siCltc^ was injected prior to DiR-labeled Exo^CTP^, liver and spleen were imaged by an *ex vivo* imaging system 6 h following injection. Of note, a much weaker DiR signal was observed in both organs by Exo^siCltc^ pre-treatment (−24.64%, *P* < 0.001 for liver; −36.74%, *P* < 0.01 for spleen; Figure 7B), indicating compromised MPS-heavy organ entrapment. Consequently, Exo^siCltc^ pre-administration effectively elevated the concentration of therapeutic Exo^CTP^ in peripheral blood as well as in ischemic hearts (Figure 7C and D). Collectively, our study demonstrated that a two-step drug delivery strategy substantially blocked hepatic and splenic MPS endocytosis, thereby facilitating the efficient delivery of therapeutic exosomes to the heart.

### Exo^siCltc^ pre-treatment amplifies cardio-protective effect of Cur@Exo^CTP^

**Figure 8.**
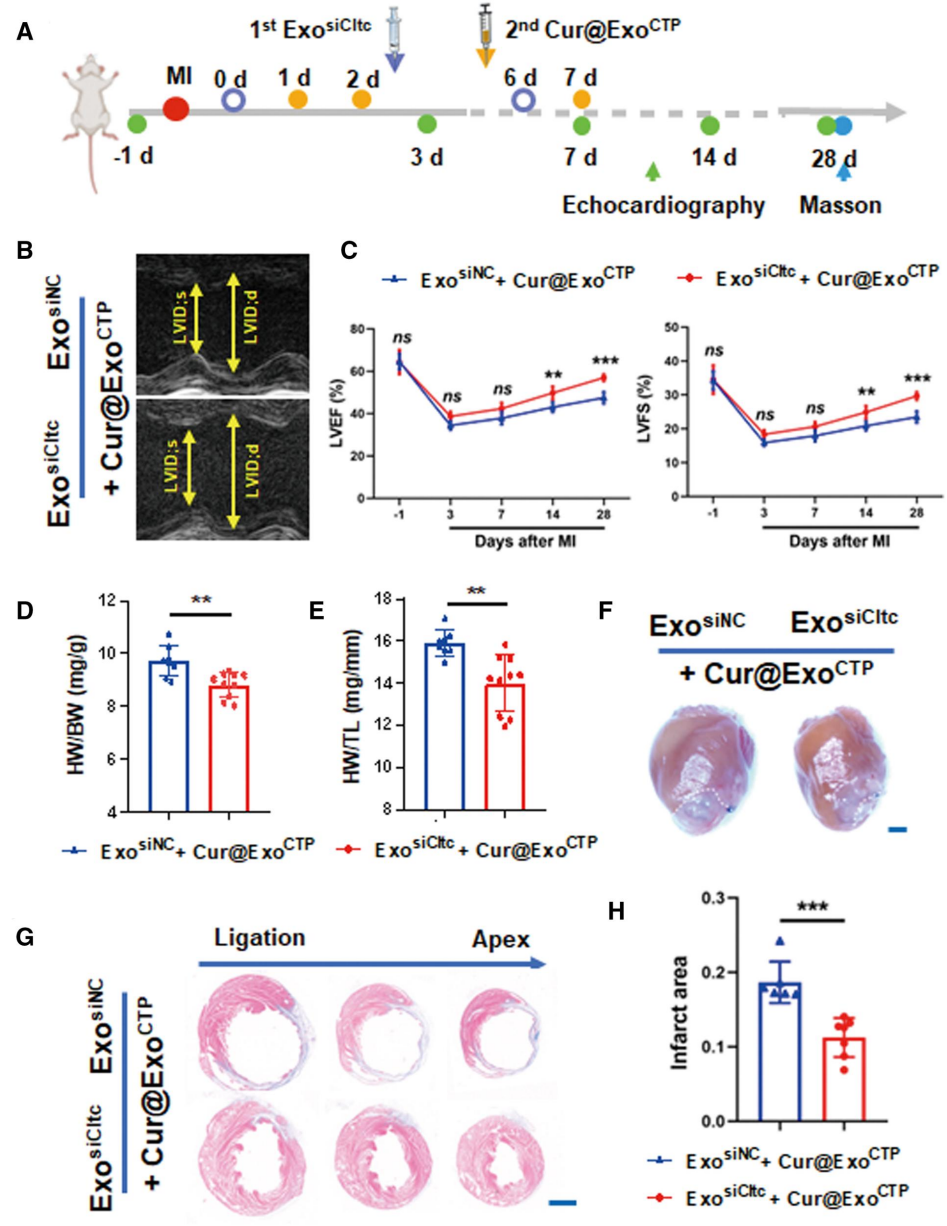
Pre-injection of Exo^siCltc^ extends cardio-protective effect of Cur@Exo^CTP^. (**A**) Schematic diagram of experimental time points. (**B**) Representative M-mode echocardiographic images with indicated treatments. (**C**) LVEF and LVFS in indicated groups (*n *=* *8–10). (**D–F**) HW/BW (**D**), HW/TL (**E**), and heart whole-view (**F**) on Day 28 post-MI (*n *=* *8–10). Scale bar, 2 mm. (**G**, **H**) Masson's staining images and quantification of infarct size on Day 28 (*n *=* *6–7). Scale bar, 2 mm. All data are represented as mean ± SD. Two-way ANOVA followed with Tukey’s post hoc test or two-tailed unpaired Student’s *t* test was utilized. *ns*: not significant, **P < 0.01, ***P < 0.001.

To further investigate cardio-protective role of our two-step delivery strategy, myocardium function, heart morphology and infarct size were evaluated on MI mice (Figure 8A). Notably, sequential administration of Exo^siCltc^ and therapeutic Cur@Exo^CTP^ effectively elicited cardiac ischemic recovery, as evidenced by optimized heart function (56.95 ± 1.61% vs. 47.52 ± 2.76%, *P* < 0.001 for LVEF; 29.68 ± 1.18% vs. 23.47 ± 1.71%, *P* < 0.001 for LVFS; Figure 8B and C), smaller heart weight (8.82 ± 0.48 vs. 9.75 ± 0.58 mg/g, *P* < 0.01 for HW/BW; 14.00 ± 1.34 vs. 15.93 ± 0.61 mg/mm, *P* < 0.01 for HW/TL; Figure 8D–F) and restricted scar formation (Figure 8G and H). In summary, our strategy, which simultaneously encouraged direct heart targeting and blocked passive liver/spleen entrapment, maximizes the therapeutic effects of encapsulated drugs.

## Conclusion

**Figure 9.**
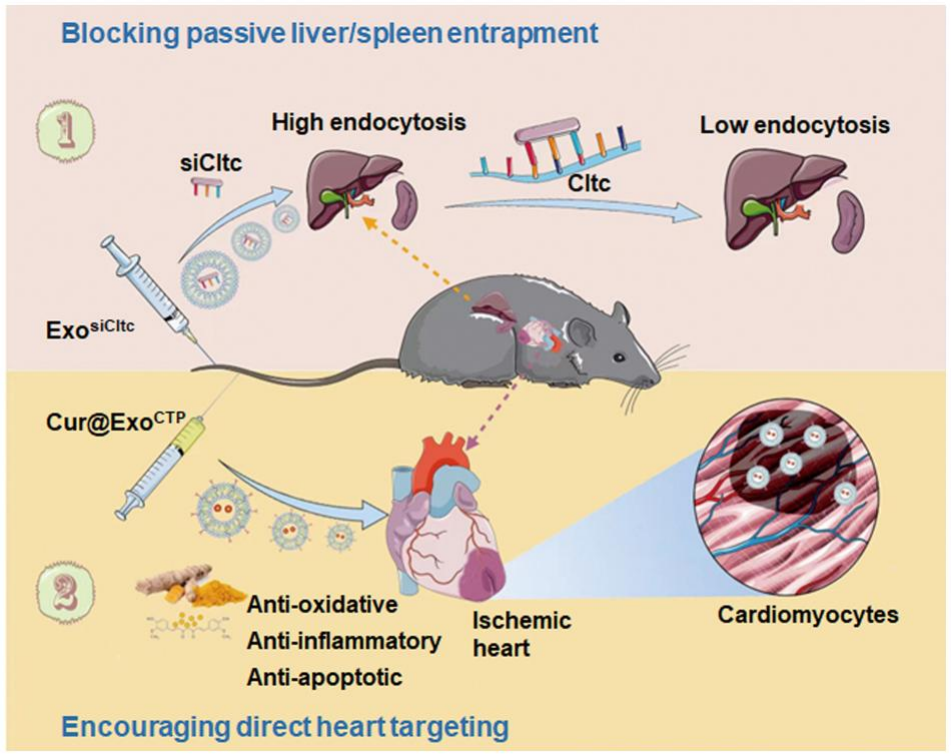
Overview of two-step drug delivery strategy benefiting ischemic wound healing.

Our research brings attention to three noteworthy discoveries. First, we presented compelling evidence demonstrating the specific targeting of modified exosomes to the ischemic heart, particularly the cardiomyocytes. Second, Exo^CTP^ augmented the accumulation and bioavailability of curcumin in the heart, ultimately leading to its superior efficacy in facilitating targeted therapy for the heart. Finally, pre-treatment with exosomes loaded with siCltc was found to effectively inhibit the endocytic function of the splenic and hepatic MPS, thereby promoting the distribution of therapeutic exosomes to the heart. In summary, we have designed a two-step drug delivery strategy intending to encourage direct heart targeting while minimizing liver and spleen entrapment, thus rendering promise for clinical translation (Figure 9).

## Supplementary Material

rbad108_Supplementary_Data
