# Supplementary material for: Precision cardiac targeting: empowering curcumin therapy through smart exosome-mediated drug delivery in myocardial infarction
Source: Regen Biomater. 2023 Dec 13;11:rbad108. doi: 10.1093/rb/rbad108 (PMC10786676; doi:10.1093/rb/rbad108)
Supplement: rbad108_Supplementary_Data [file rbad108_supplementary_data.pdf]

Precision cardiac targeting: empowering curcumin therapy through smart exosome-mediated drug delivery in myocardial infarction

Ming Chen[1, #], Shengnan Wang[1, #], Yihuan Chen[1, #], Han Shen[1], Lei Chen[1], Liang Ding[1], Qingsong Tang[1], Ziying Yang[1], Weiqian Chen[1, *], Zhenya Shen[1, *]

[1] Department of Cardiovascular Surgery of the First Affiliated Hospital & Institute for Cardiovascular Science, Medical College, Soochow University, Suzhou 215006, China.

[#] These authors contributed equally to this paper

Corresponding authors: Zhenya Shen (uuzyshen@aliyun.com) and Weiqian Chen (chenweiqian@suda.edu.cn)

Supplementary Materials

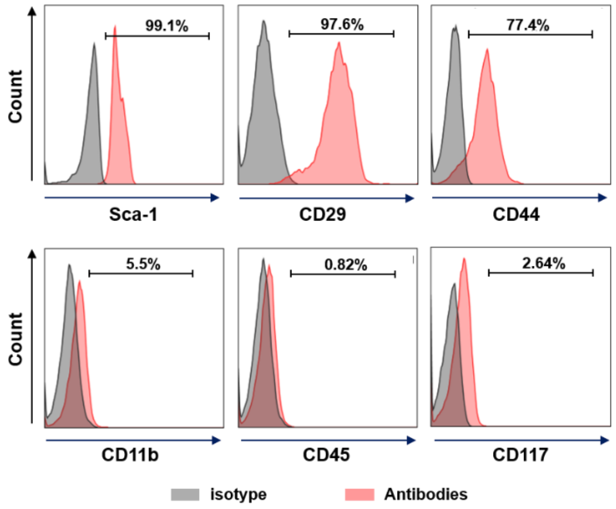

Figure S1 Expression pattern characterization for MSC surface markes, including Sca-1, CD29, CD44, CD11b, CD45, and CD117.

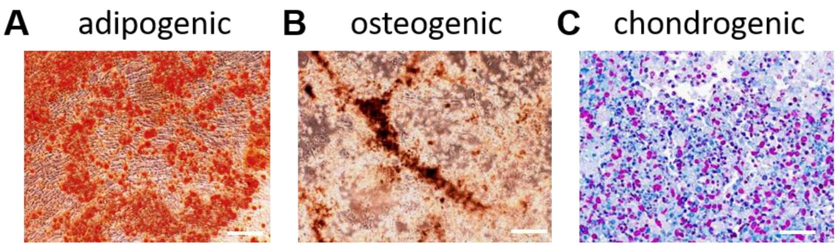

Figure S2 Representative photographs of adipogenesis (A), osteogenesis (B), and chondrogenesis (C) of MSCs. Scale bar, A, 200 μm; B, 200 μm; C, 50 μm.

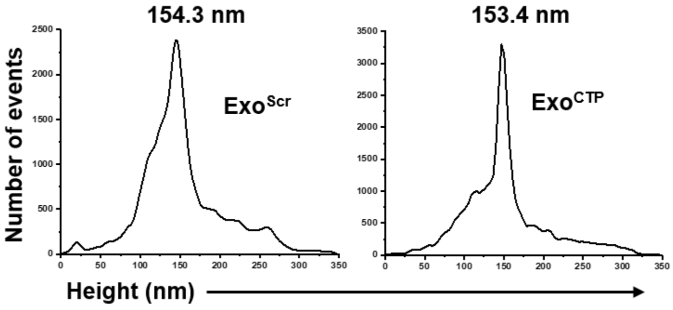

Figure S3 Particle height distribution measured by atomic force microscopy (AFM).

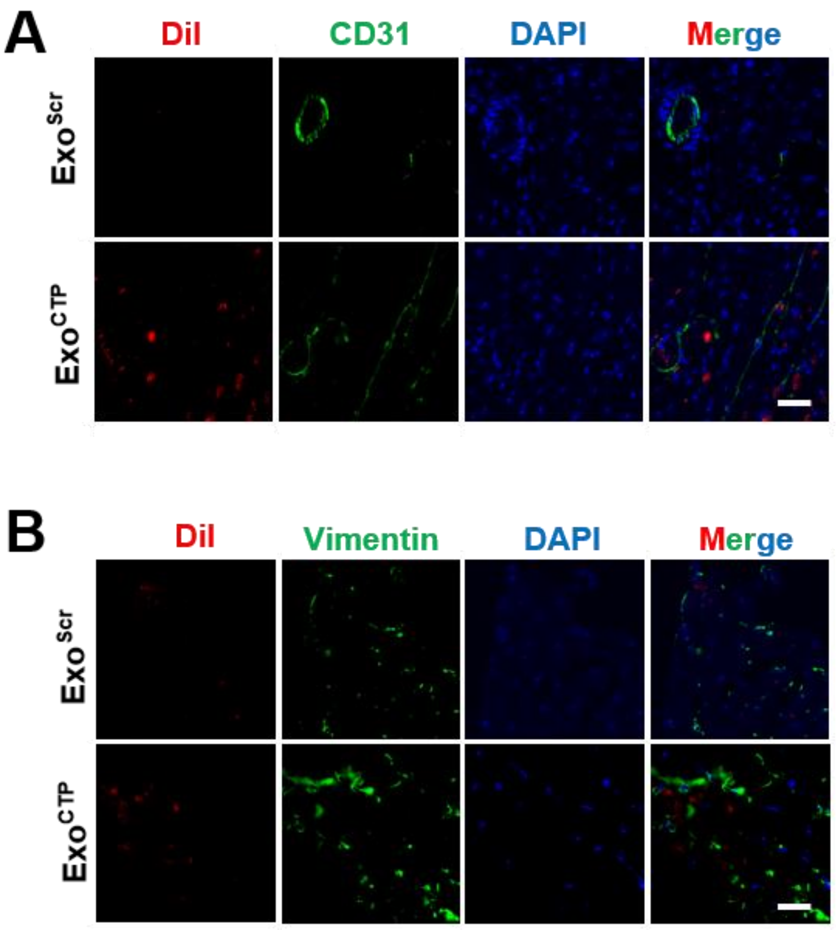

Figure S4 Representative heart section images of DiI-labeled Exo^{Scr} or Exo^{CTP} taken up by $CD31^{+}$ endothelial cells (A) and $Vimentin^{+}$ fibroblasts (B). Scale bar, 50 μm.

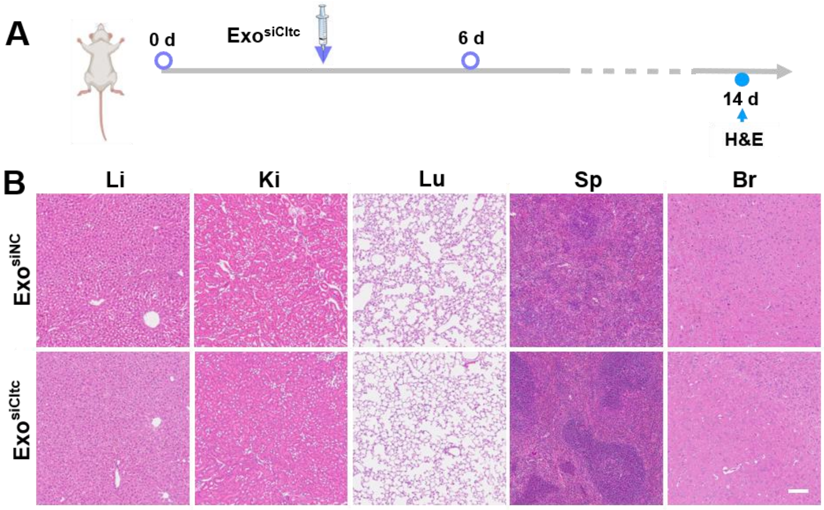

Figure S5 Safety verification of Exo^{siCltc}. (A&B) H&E staining of vital organs after indicated treatments. Scale bar, 100 μm. Li, liver; Ki, kidney; Lu, lung; Sp, spleen; Br, brain.
